# Enhanced proliferation of pancreatic acinar cells in MRL/MpJ mice is driven by severe acinar injury but independent of inflammation

**DOI:** 10.1038/s41598-018-27422-0

**Published:** 2018-06-20

**Authors:** Marta Bombardo, Ermanno Malagola, Rong Chen, Arcangelo Carta, Gitta M. Seleznik, Andrew P. Hills, Rolf Graf, Sabrina Sonda

**Affiliations:** 1 Swiss Hepato-Pancreato-Biliary Center, Department of Visceral and Transplantation Surgery, University Hospital, Zurich, Switzerland; 2 Center for Integrative Human Physiology (ZIHP), University of Zurich, Zurich, Switzerland; 3 School of Health Sciences, College of Health and Medicine, University of Tasmania, Tasmania, Australia

## Abstract

Adult pancreatic acinar cells have the ability to re-enter the cell cycle and proliferate upon injury or tissue loss. Despite this mitotic ability, the extent of acinar proliferation is often limited and unable to completely regenerate the injured tissue or restore the initial volume of the organ, thus leading to pancreatic dysfunction. Identifying molecular determinants of enhanced proliferation is critical to overcome this issue. In this study, we discovered that Murphy Roths Large (MRL/MpJ) mice can be exploited to identify molecular effectors promoting acinar proliferation upon injury, with the ultimate goal to develop therapeutic regimens to boost pancreatic regeneration. Our results show that, upon cerulein-induced acinar injury, cell proliferation was enhanced and cell cycle components up-regulated in the pancreas of MRL/MpJ mice compared to the control strain C57BL/6. Initial damage of acinar cells was exacerbated in these mice, manifested by increased serum levels of pancreatic enzymes, intra-pancreatic trypsinogen activation and acinar cell apoptosis. In addition, MRL/MpJ pancreata presented enhanced inflammation, de-differentiation of acinar cells and acinar-to-ductal metaplasia. Manipulation of inflammatory levels and mitogenic stimulation with the thyroid hormone 5,3-L-tri-iodothyronine revealed that factors derived from initial acinar injury rather than inflammatory injury promote the replicative advantage in MRL/MpJ mice.

## Introduction

Recovery from mild forms of pancreatitis, an inflammatory disease of the exocrine pancreas, occurs via regeneration of pancreatic acinar cells. However, the extent of the regenerative process is limited and pancreatic injuries are often associated with inability to replace lost or mal-functioning acinar cells, thus resulting in pancreatic dysfunction and pancreatic insufficiency^1^. In this context, the identification of mouse strains with elevated regenerative ability would provide a useful platform to identify the molecular components able to improve pancreatic regeneration.

In this study, we investigated whether enhanced pancreatic regeneration is present following induction of pancreatitis in the Murphy Roths Large (MRL/MpJ) mouse strain. These mice are characterized by a striking ability of epimorphic regeneration in several adult organs and tissues, first described by Heber-Katz and colleagues^2^. Epimorphic regeneration is normally found during embryonic development^3,4^, while the typical default response to injury in adult mammals results in the development of an inflammatory reaction and formation of scar tissue. In this respect, MRL/MpJ mice provide a unique opportunity to understand the molecular mechanisms that differentiate regeneration from a simple repair process.

The extensive body of evidence to date to elucidate these mechanisms revealed that the “super healing” attribute is multigenic^5–10^. Interestingly, a common feature that emerged from transcriptomic studies is that the healing process in different regenerating tissues of the MRL/MpJ mice is associated with repression of genes responsible for inflammation, a regulation also typical of neonatal regeneration^6^, and with a different composition of secreted inflammatory molecules. Noteworthy is that, while the altered basal immune system and immune response to injury observed in MRL/MpJ mice seems to be beneficial in the regeneration of selected tissues, these mice develop an autoimmune phenotype in the late stage of life^11,12^. A direct correlation between “super healing” and autoimmunity is still a matter of debate, but the existence of autoimmunity-prone mouse strains without epimorphic regeneration ability suggests a lack of causality between these two phenotypes^13^.

So far, enhanced regenerative potential and reduced inflammation have been found in several injury models in MRL/MpJ mice compared with control “non-healer” C57BL/6 mice, including ear hole punches, heart injury, digit amputation, alkali-burned cornea, spinal cord injury and articular fracture (reviewed in^14^ and^15,16^). Here we investigated whether enhanced regeneration is also present following inflammatory injury of pancreatic acinar cells. To this aim, we compared the pathophysiological responses in MRL/MpJ mice and in the control strain C57BL/6 following administration of cerulein, the most widespread experimental approach to induce acinar cell damage and pancreatic inflammation^17^. As male MRL/MpJ mice develop spontaneous autoimmune pancreatic inflammation at a late life stage (40% incidence in 46–50 week old mice^18^), we chose to analyze only adult animals of 8–14 weeks of age. In this way, the obtained results are more likely to reflect intrinsic differences between the two strains, without the confounding aspect of autoimmune manifestations.

## Results

### Cell proliferation is enhanced in MRL/MpJ mice following cerulein administration

**Figure 1:**
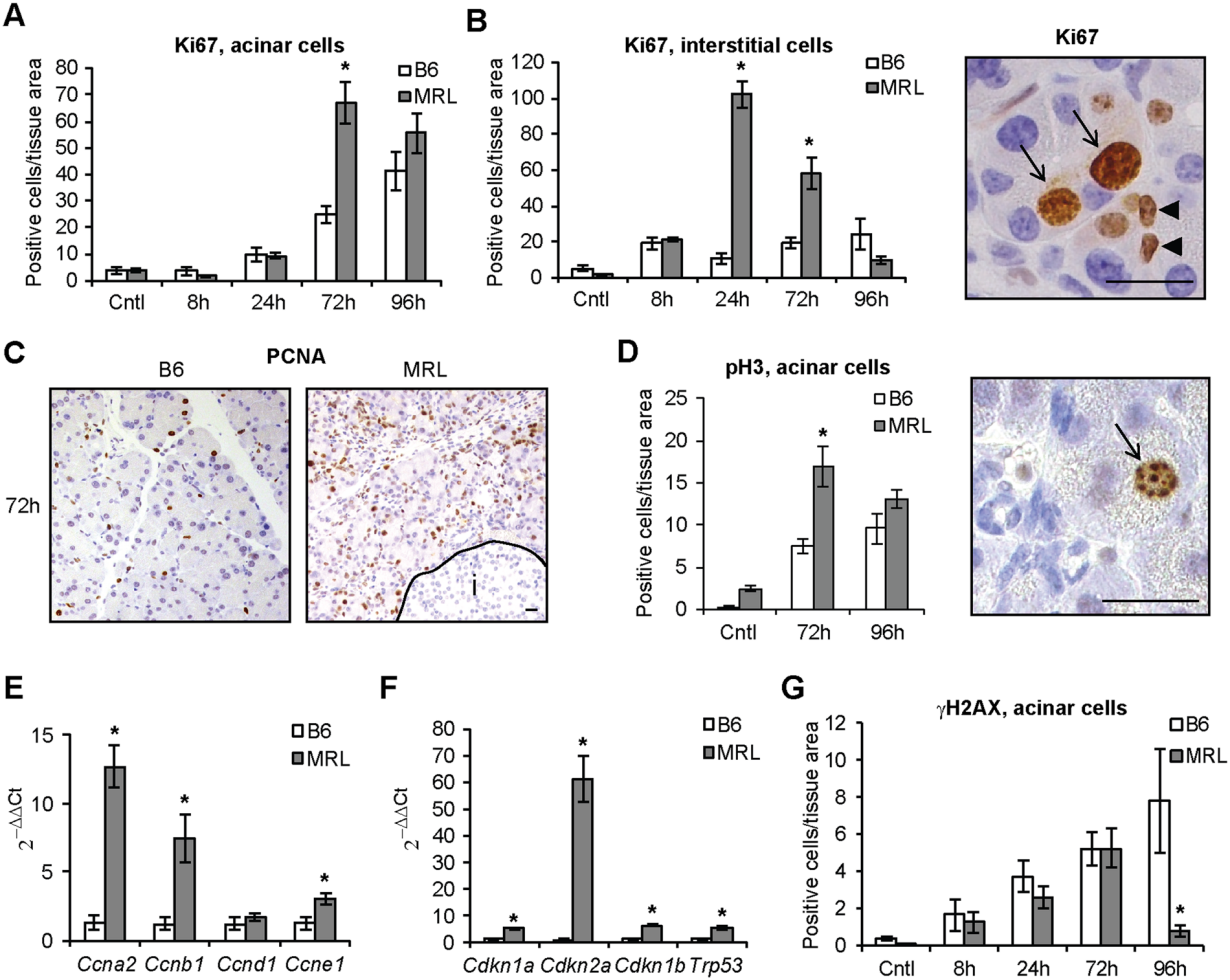
Cell proliferation is enhanced in MRL/MpJ mice following cerulein administration. Quantification of Ki67-positive proliferating acinar (**A**) and interstitial (**B**) cells in C57BL/6 (B6) and MRL/MpJ (MRL) mice in control (Cntl) animals and at the indicated time after cerulein administration. Right panel, representative microphotograph of stained acinar (arrows) and interstitial (arrowheads) cells. (**C**) Proliferating cell nuclear antigen (PCNA) staining in pancreata 72 h after cerulein administration. I, islet of Langerhans. (**D**) Quantification of phospho-histone 3 (pH3)-positive proliferating acinar cells in control (Cntl) animals and at the indicated time after cerulein administration. Arrow indicates a stained acinar cell. qPCR of cyclins (**E**) and cell cycle inhibitors (**F**) in pancreata 72 h after cerulein administration. (**G**) Quantification of DNA damage in acinar cells in control (Cntl) animals and at the indicated time after cerulein administration. Results are average ± SEM (n = 5), *P < 0.05. Scale bars: 25 μm.

To test whether pancreatic acinar proliferation was enhanced in MRL/MpJ mice, we induced acinar cell injury by cerulein administration at supra-physiological concentration, the most common experimental approach to trigger cell proliferation and development of inflammation^17^. Untreated 8–14 weeks old MRL/MpJ mice, which do not show symptoms of autoimmune pancreatitis nor produce autoantibodies (Fig. S1) did not present spontaneous activation of cell cycle in pancreatic cells compared with C57BL/6 animals (Fig. 1A,B). However, increased gene expression of selected members of cyclins, critical cellular components that promote cell cycle progression by activating cyclin*-*dependent kinases (CDKs), CDK inhibitor p27 and p53 was observed in untreated mice (Fig. S2).

Upon cerulein treatment, MRL/MpJ mice displayed faster activation of cell proliferation, as seen by higher number of both acinar (Fig. 1A) and interstitial (Fig. 1B) cells positive for the general cell cycle activation marker Ki67. The proliferative advantage of MRL/MpJ acinar cells was most evident 72 h after cerulein administration, and detected upon staining with the S phase marker proliferating nuclear antigen (PCNA) (Fig. 1C) and the mitosis marker phospho-histone 3 (pH3) (Fig. 1D).

The proliferative advantage observed at the 72 h time point was associated with increased pancreatic expression of cyclins (Fig. 1C), CDK inhibitors and p53 (Fig. 1D), while levels of DNA damage did not increase compared with C57BL/6 (Fig. 1E). This is of particular interest as selective down-regulation of the CDKi p21^WAF1/Cip1^ and increased DNA damage were causally linked to increased proliferation in MRL/MpJ dermal skin cells^19^. Collectively, these data indicate that increased acinar proliferation observed in MRL/MpJ mice following cerulein administration is not mechanistically associated with down-regulation of cell cycle checkpoints or activation of DNA damage response.

### Acinar cell damage is enhanced in MRL/MpJ mice following cerulein administration

**Figure 2:**
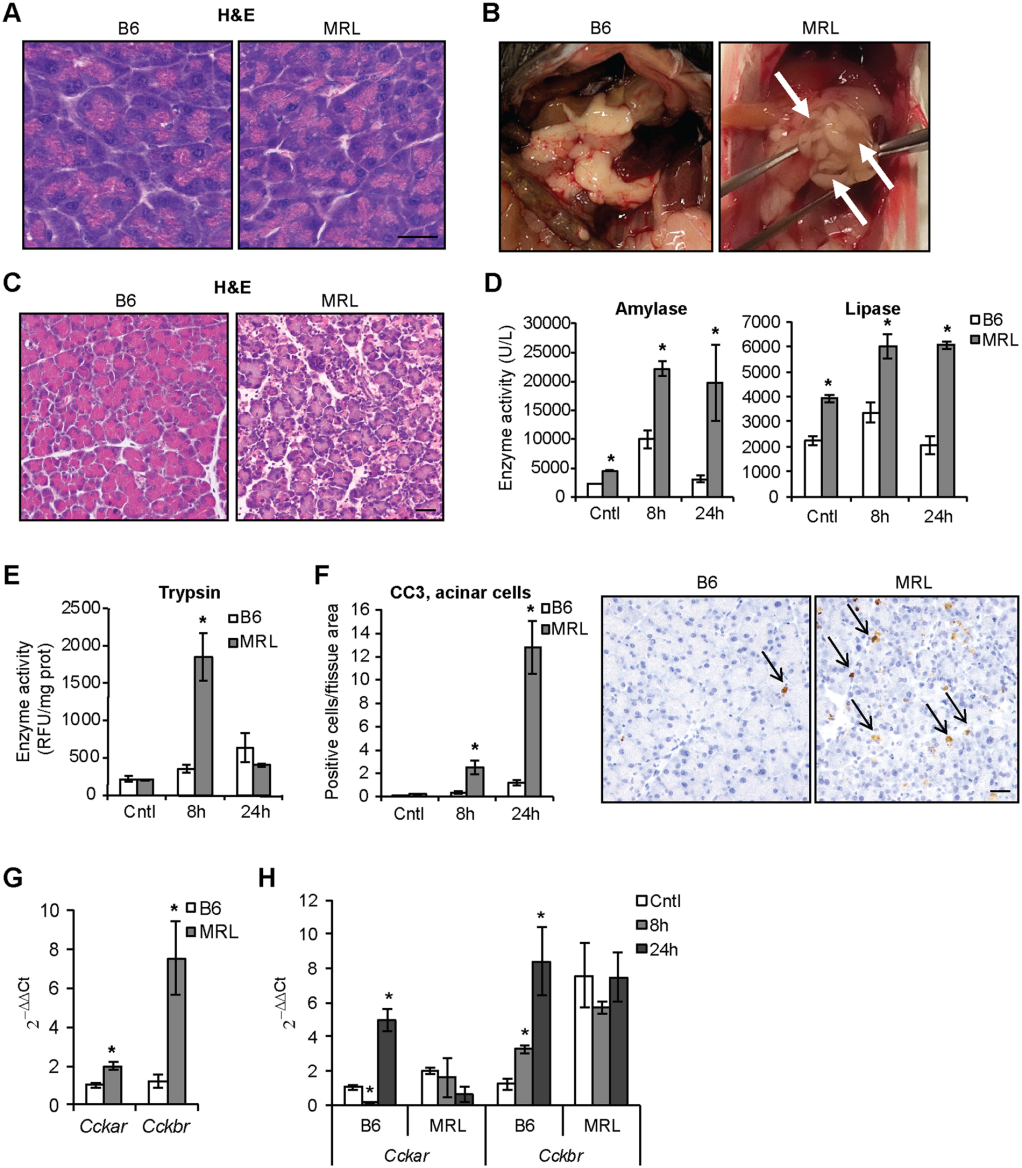
Acinar cell damage is enhanced in MRL/MpJ mice following cerulein administration. (**A**) Hematoxylin and Eosin (H&E) staining of untreated pancreata of C57BL/6 (B6) and MRL/MpJ (MRL) mice. (**B**) Macroscopic appearance of pancreata 24 h after cerulein administration. Note the prominent edema (arrows) in MRL samples. (**C**) Hematoxylin and Eosin (H&E) staining of pancreata 24 h after cerulein administration. (**D**) Serum levels of amylase and lipase enzymatic activity in control (Cntl) animals and at the indicated time following cerulein administration. (**E**) Pancreatic levels of trypsin activity in control (Cntl) animals and at the indicated time following cerulein administration. (**F**) Quantification of cleaved caspase 3 (CC3)-positive apoptotic acinar cells in control (Cntl) animals and at the indicated time following cerulein administration. Right panels, representative micrographs of CC3-positive cells 24 h following cerulein administration. (**G**) qPCR of CCK receptors in pancreata of untreated mice. (**H**) qPCR of CCK receptors in pancreata in control (Cntl) animals and at the indicated time following cerulein administration. Results are average ± SEM (n = 5), *P < 0.05. Scale bars: 50 μm.

Prompted by the increased cell proliferation observed in MRL/MpJ mice, we then investigated whether these mice were more sensitive to cerulein administration and thus developed an increased acinar cell damage compared with C57BL/6 animals. In the untreated condition, pancreatic parenchyma was histologically undistinguishable in the two strains (Fig. 2A). However, increased tissue damage in the form of pronounced edema was evident macroscopically (Fig. 2B) and histologically (Fig. 2C) in MRL/MpJ mice 24 h after cerulein administration. Furthermore, blood levels of amylase and lipase enzymatic activities, early diagnostic signs of acinar cell damage, were more pronounced and long lasting in MRL/MpJ mice, whereas these parameters returned to baseline levels in C57BL/6 animals already after 24 h of cerulein treatment (Fig. 2D). Increased acinar cell damage in MRL/MpJ mice was further confirmed by increased trypsinogen activation into trypsin (Fig. 2E) and increased acinar cell apoptosis, quantified by cleaved caspase 3 staining (Fig. 2F). Serum levels of glutamate pyruvate transaminase (GPT) and glutamic oxaloacetic transaminase (GOT) enzymatic activities, which increase during inflammatory damage of the pancreas^20,21^, were higher in MRL/MpJ animals (Fig. S3A). Conversely, lung inflammation, assessed by myeloperoxidase activity, was comparable in the two strains (Fig. S3B). Collectively, these observations indicate that MRL/MpJ mice develop a more severe damage of acinar cells following cerulein administration compared with the control strain C57BL/6.

Finally, as cerulein acts on acinar cells via stimulation of cholecystokinin (CCK) receptors, we assessed whether higher acinar damage was associated with higher CCK receptor expression. Expression of both CCK receptors *Cckar* and *Cckbr* was elevated in MRL/MpJ pancreata compared with C57BL/6 animals in untreated condition (Fig. 2G) and presented different regulation during cerulein administration (Fig. 2H).

### Development of inflammation is enhanced in MRL/MpJ mice following cerulein administration

**Figure 3:**
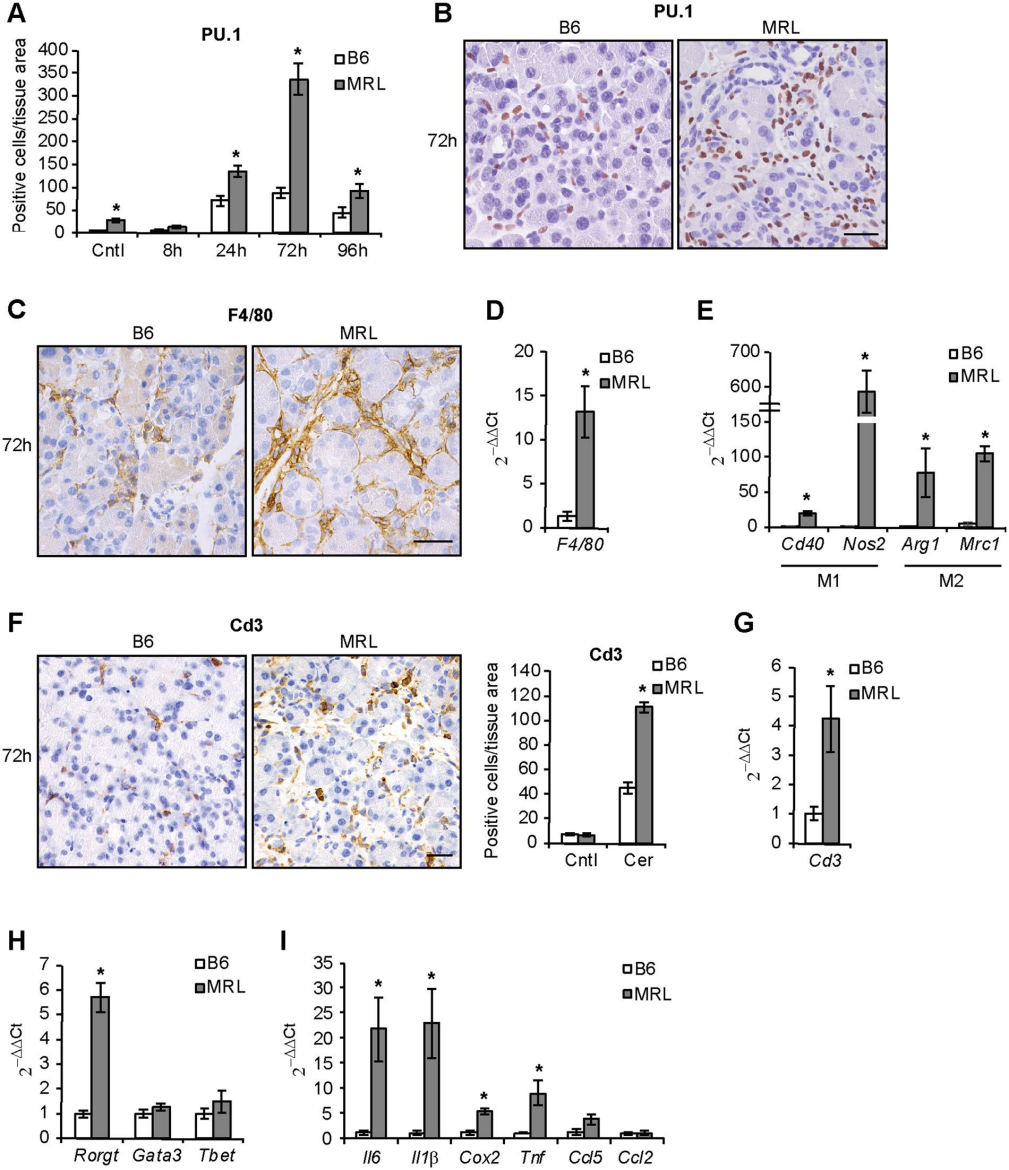
Pancreatic inflammation is enhanced in MRL/MpJ mice following cerulein administration. (**A**) Quantification of PU.1-positive leukocytes infiltrating the pancreas of C57BL/6 (B6) and MRL/MpJ (MRL) mice in control (Cntl) animals and at the indicated time after cerulein administration. (**B**) Immunostaining of PU.1-positive leukocytes infiltrating the pancreas 72 h after cerulein administration. Immunostaining (**C**) and qPCR (**D**) of F4/80-positive macrophages infiltrating the pancreas 72 h after cerulein administration. (**E**) qPCR of M1 and M2 macrophage markers 72 h after cerulein administration. Quantification of immunostaining (**F**) and qPCR (**G**) of Cd3-positive T cells infiltrating the pancreas 72 h after cerulein administration. (**H**) qPCR of Th-cell differentiation markers. (**I**) qPCR of pro-inflammatory factors 72 h after cerulein administration. Results are average ± SEM (n = 5), *P < 0.05. Scale bars: 50 μm.

Next, we quantified whether increased cerulein-induced acinar cell damage resulted in exacerbated inflammatory reaction in the pancreas of MRL/MpJ mice. The evaluation of inflammation is of particular interest in the context of acinar proliferation as the increased regenerative ability observed previously in different MRL/MpJ tissues was found to be associated with repression of inflammatory reaction^6,14,22^. In untreated conditions, MRL/MpJ pancreata did not show any alteration in the expression of inflammatory chemokines (Fig. S4A), cytokines (Fig. S4B), markers of macrophages and T cells (Fig. S4C), or T cell transcription factors (Fig. S4D). However, contrary to previous reports, we detected increased infiltration of PU.1-positive leukocytes in MRL/MpJ pancreata, peaking 72 h after cerulein administration and decreasing in both strains 96 h after the treatment (Fig. 3A,B). Given the transient nature of leukocyte infiltration, we chose to investigate further the inflammatory components at 72 h of treatment, a time point when the differences between the two strains are more pronounced. Amongst the various leukocyte populations, macrophage infiltration was significantly higher in MRL/MpJ pancreata, as determined by immunohistochemistry (Fig. 3C) and gene expression analyses (Fig. 3D) of F4/80 expression. Markers of both classical (*Cd40, Nos2*) and alternative (*Arg1, Mrc1*) macrophage activation were elevated in MRL/MpJ mice (Fig. 3E). Similarly, levels of Cd3 positive T cells (Fig. 3F,G) were higher in pancreata of these mice. Amongst the transcription factors orchestrating the differentiation of T cells, *Rorgt* was expressed more in MRL/MpJ mice (Fig. 3H). In parallel with increased leukocyte infiltration, levels of pro-inflammatory mediators were also higher in MRL/MpJ mice (Fig. 3I). These data suggest that MRL/MpJ mice develop a more severe pancreatic inflammation than control C57BL/6 animals following cerulein treatment.

### ADM is enhanced in MRL mice following cerulein administration

**Figure 4:**
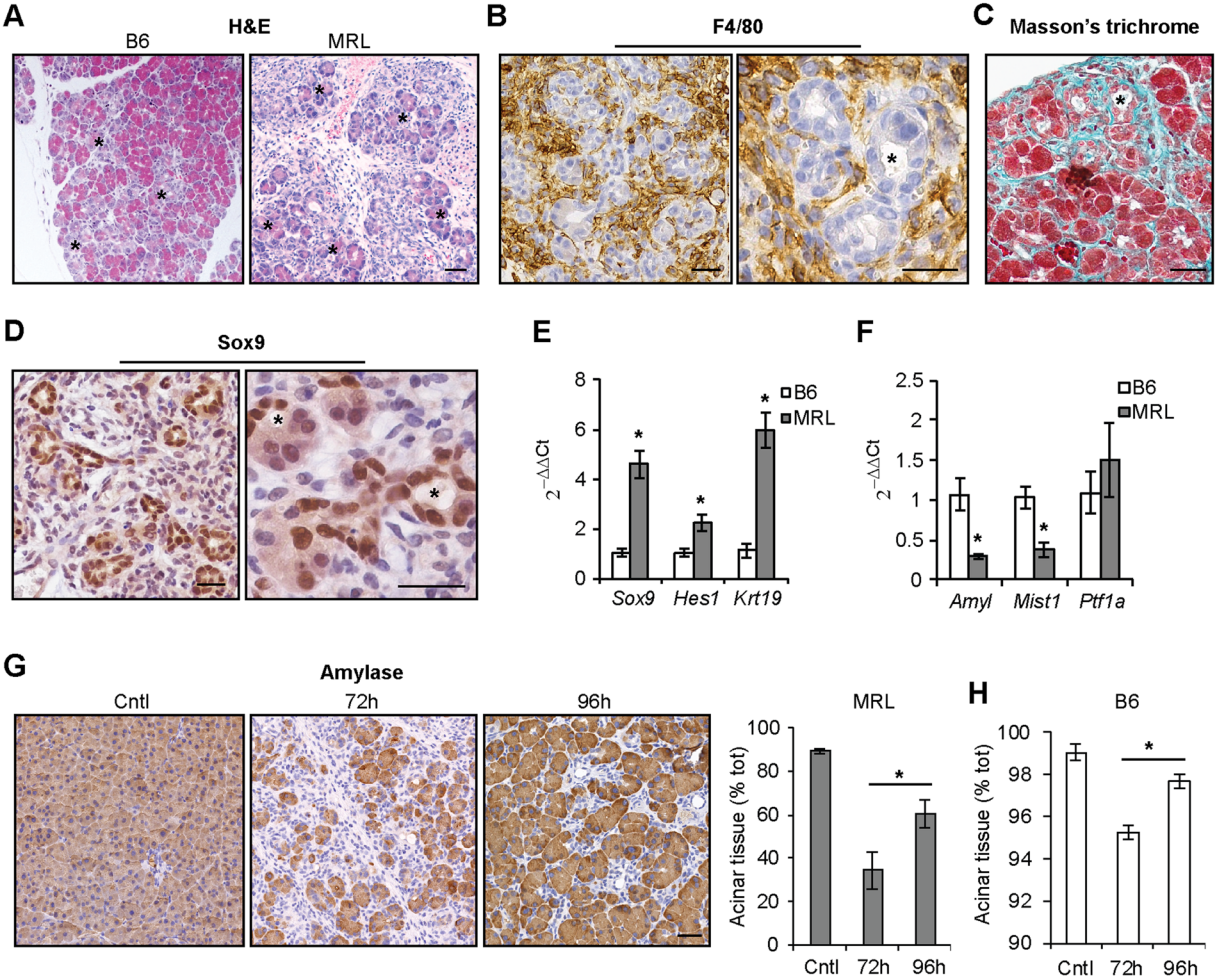
ADM is enhanced in MRL/MpJ mice following cerulein administration. (**A**) Hematoxylin and Eosin (H&E) staining of pancreata of C57BL/6 (B6) and MRL/MpJ (MRL) mice 72 h after cerulein administration. Asterisks indicate area with cytosolic discoloration of acinar cells. Staining of F4/80-positive macrophages (**B**), collagen (Masson’s trichrome) (**C**) and Sox9 (**D**) in ADM areas of MRL/MpJ mice 72 h after cerulein administration. Asterisks indicate formation of tubular structures. qPCR of progenitor markers (**E**) and differentiation markers (**F**) in pancreata 72 h after cerulein administration. (**G**) Quantification of intact acinar tissue positive for amylase in MRL/MpJ mice in control (Cntl) animals and at the indicated time after cerulein administration. (**H**) Quantification of intact acinar tissue positive for amylase in B6 mice in control (Cntl) animals and at the indicated time after cerulein administration. Results are average ± SEM (n = 5), ^*^P < 0.05. Scale bars: 50 μm.

Pancreatic recruitment of inflammatory cells, especially macrophages, supports the transient de-differentiation of acinar cells and acinar-to-ductal metaplasia (ADM)^23^. In this process, acinar cells lose their differentiated phenotype, manifested histologically by cytosolic discoloration of acinar cells upon Hematoxylin and Eosin staining (Fig. 4A, asterisk) and formation of tubular structures (Fig. 4B,C asterisks). This process is accompanied at the transcriptional level by up-regulation of progenitor-like genes and down-regulation of differentiation genes, including digestive enzymes and associated transcription factors’ expression^24^. Extended areas of ADM were present in MRL/MpJ pancreata 72 h after cerulein administration (Fig. 4A), the time point when the proliferative advantage of acinar cells was evident. ADM showed the typical features of prominent stromal reaction with infiltrating F4/80-positive macrophages (Fig. 4B), collagen deposition (Fig. 4C), and nuclear expression of the progenitor-like marker Sox9 (Fig. 4D). Moreover, transcript levels of progenitor-like genes were prominently up-regulated in MRL/MpJ mice 72 h after cerulein treatment (Fig. 4E), while they were comparable to the control strain in untreated conditions (Fig. S5A). Consistent with increased de-differentiation of acinar cells 72 h after cerulein administration, MRL/MpJ pancreata showed transcript down-regulation of differentiation genes (Fig. 4F). In untreated conditions, amylase levels were comparable in the two strains, while Mist1 and Ptf1 transcript were up-regulated MRL/MpJ mice (Fig. S5B).

ADM formation is intimately connected with pancreatic regeneration, as transient ADM may support pancreatic regeneration^17^, while persistent ADM is indicative of impaired regeneration. To test whether ADM resolved and acinar tissue regenerated in MRL/MpJ mice, we quantified the amount of intact acinar tissue positive for the enzyme amylase at 72 h and 96 h following cerulein administration. The analysis revealed that the percentage of amylase-positive acinar tissue decreased 72 h after induction of pancreatitis, concomitantly with ADM formation, but it increased afterward at 96 h (Fig. 4G). Quantification of amylase-positive tissue in C57BL/6 mice is shown as a comparison in Fig. 4H, highlighting the reduced amount of compromised acinar tissue, which correlates with the extent of ADM normally reported in the range of few percent of total tissue area.

Collectively, these data suggest that, despite the major extent of ADM observed in MRL/MpJ mice, ADM is also transient in this strain and pancreatic regeneration progresses over time.

### Acinar cell proliferation is comparable in MRL/MpJ and C57BL/6 mice upon mitogenic stimulation

**Figure 5:**
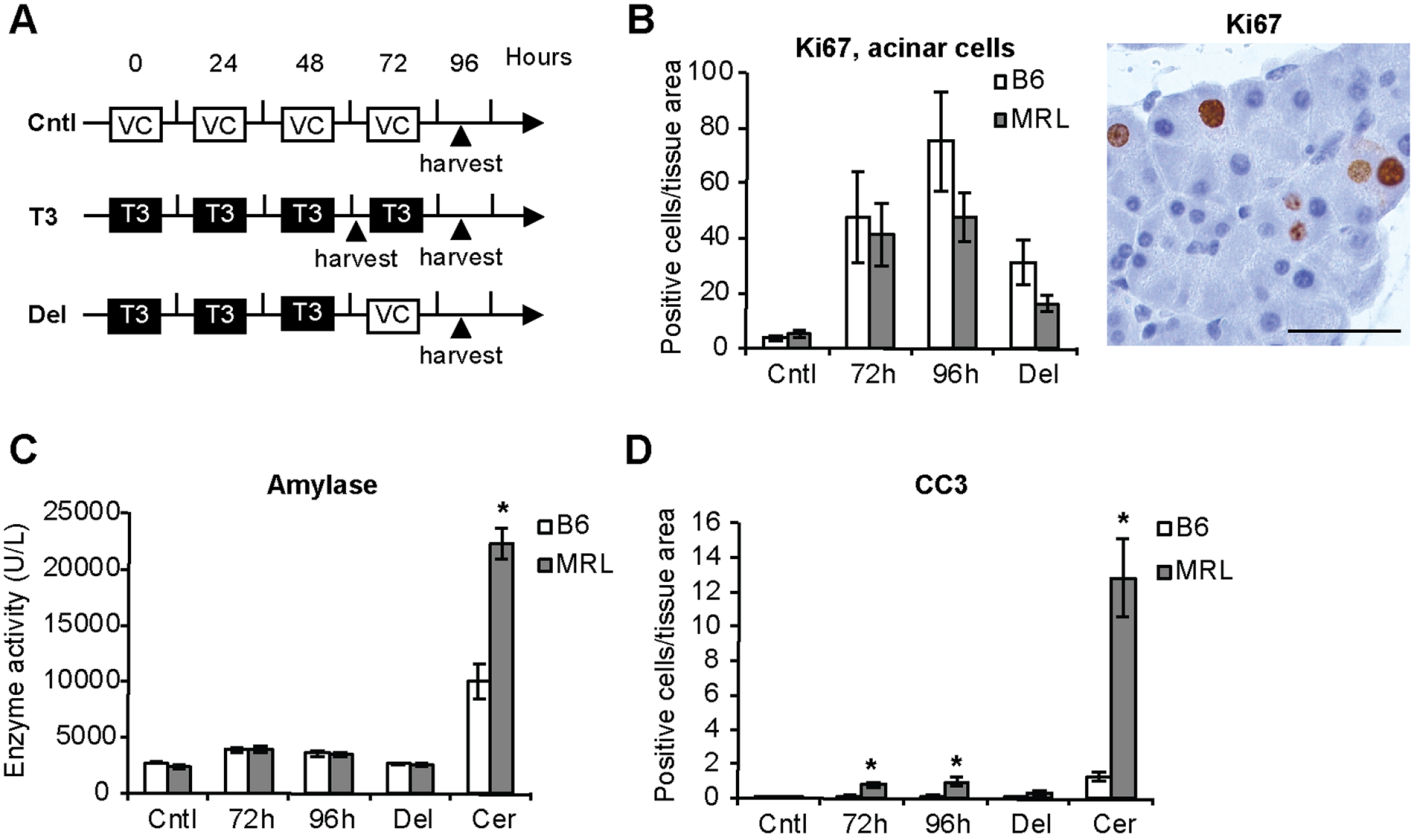
Cell proliferation is comparable in MRL/MpJ and C57BL/6 control mice following mitogenic stimulation. (**A**) Schematic representation of 3,5,3-L-tri-iodothyronine (T3) treatment. VC, vehicle control. Cntl, control animals. Del, delayed model. (**B**) Quantification of Ki67-positive replicating acinar cells upon T3 administration. Right panel, representative microphotograph of stained acinar cells. (**C**) Serum levels of amylase enzymatic activity in control (Cntl) animals and at the indicated time following T3 administration. Amylase enzymatic activity levels following cerulein-induced damage (Cer) are shown as a comparison. (**D**) Quantification of cleaved caspase 3 (CC3)-positive apoptotic acinar cells at the indicated time following T3 administration. CC3 levels following cerulein-induced damage (Cer) are shown as a comparison. Results are average ± SEM (n = 5), *P < 0.05. Scale bars: 50 μm.

Our data showed that increased cell proliferation in MRL/MpJ mice correlates with increased acinar damage and increased pancreatic inflammation, thus raising the question whether the proliferative advantage in these mice may be a consequence of these factors. To test whether increased acinar cell damage is the underlining cause for increased acinar proliferation, we quantified the latter upon mitogenic stimulation with the thyroid hormone 3,5,3-L-tri-iodothyronine (T3), which acts as a mitogen for pancreatic acinar cells^25,26^, and its action does not depend on tissue damage. For this aim, we compared acinar proliferation in the two strains using two approaches: i) We analyzed whether acinar proliferation was time-dependent, by harvesting mice at 72 h and 96 h after daily T3 administration. ii) In addition, we analyzed whether acinar cell proliferation was long lasting upon removal of the mitogenic stimulus, by harvesting mice at 96 h after receiving T3 only in the first three days, a so-called delayed model. T3 treatment schemes are depicted in Fig. 5A. T3-treated MRL/MpJ and C57BL/6 mice revealed similar acinar proliferation in all the regimens tested (Fig. 5B). Importantly, evaluation of tissue injury showed that serum levels of amylase (Fig. 5C) and lipase (Fig. S6) enzymatic activities were comparable in the two strains. In addition, levels of acinar apoptosis following T3 treatment were well below the levels detected during cerulein-induced damage (Fig. 5D). Collectively, these results show that MRL/MpJ acinar cells do not have an overt proliferative advantage over the C57BL/6 strain during mitogenic stimulation with T3 devoid of acinar damage.

### Pancreatic cell proliferation does not decrease in MRL/MpJ mice upon reduction of inflammation

**Figure 6:**
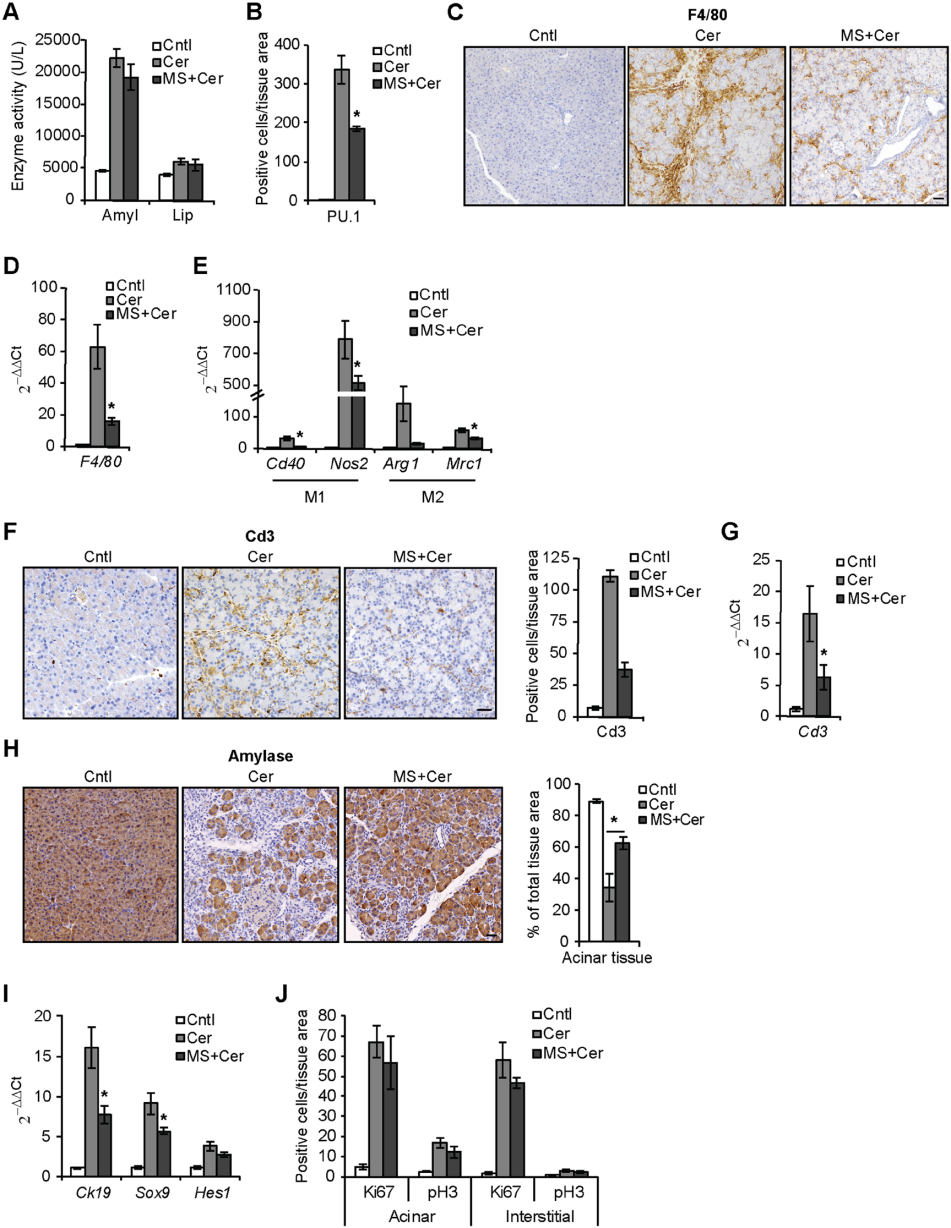
MS-275 treatment reduces pancreatic inflammation and ADM but not cell proliferation in MRL/MpJ mice. (**A**) Serum levels of amylase (Amyl) and lipase (Lip) enzymatic activity 8 h after cerulein administration in the presence of MS-275 (MS). Cntl, control animals. (**B**) Quantification of PU.1-positive leukocytes infiltrating the pancreas in control (Cntl) animals and 72 h after cerulein administration in the presence of MS-275. Immunostaining (**C**) and qPCR (**D**) of F4/80-positive macrophages infiltrating the pancreas in control (Cntl) animals and 72 h after cerulein administration in the presence of MS-275. (**E**) qPCR of M1 and M2 macrophage markers in control (Cntl) animals and 72 h after cerulein administration in the presence of MS-275. Immunostaining quantification (**F**) and qPCR (**G**) of Cd3-positive T cells infiltrating the pancreas in control (Cntl) animals and 72 h after cerulein administration in the presence of MS-275. (**H**) Quantification of intact acinar tissue positive for amylase in control (Cntl) animals and 72 h after cerulein administration in the presence of MS-275. (**I**) qPCR of progenitor markers in control (Cntl) animals and 72 h after cerulein administration in the presence of MS-275. (**J**) Quantification of Ki67 and pH3-positive proliferating acinar and interstitial cells in control (Cntl) animals and 72 h after cerulein administration in the presence of MS-275. Results are average ± SEM (n = 5), *P < 0.05. Scale bars: 50 μm.

Finally, to dissect the contribution of inflammation in promoting acinar proliferation, we treated MRL/MpJ mice with MS-275, a selective inhibitor of the class I histone deacetylases (HDACs) that effectively reduces pancreatic inflammation triggered by cerulein administration in C57BL/6 mice^27^. Similar to what was previously observed in the control strain, MS-275 treatment did not decrease the initial acinar cell damage in MRL/MpJ mice, as shown by comparable blood levels of pancreatic enzyme activity 8 h after induction of pancreatitis (Fig. 6A). However, MS-275 treatment robustly decreased pancreatic infiltration of PU.1-positive leukocytes (Fig. 6B), levels of F4/80-positive macrophages (Fig. 6C,D), expression of macrophage polarization markers (Fig. 6E), and amount of Cd3-positive T cells (Fig. 6F,G) 72 h after cerulein administration. Concomitant with the reduction of inflammatory cell infiltration, MS-275 also increased the amount of intact acinar tissue expressing amylase (Fig. 6H) and the expression of progenitor-like markers (Fig. 6I) compared with cerulein-treated mice. Of note, similar phenotypes of reduced inflammation and better tissue preservation consequent MS-275 treatment were previously observed in C57BL/6 animals^23^, suggesting that the levels of pancreatic inflammation supports the development of ADM in both strains.

Importantly, despite the reduced inflammation observed upon MS-275 treatment, proliferation of pancreatic cells did not decrease in the presence of the inhibitor, as quantified by Ki67 and pH3 expression (Fig. 6J). This suggests that the enhanced proliferation observed in pancreatic MRL/MpJ cells does not depend on the higher levels of inflammatory cell infiltration observed in these animals.

## Discussion

The MRL/MpJ mouse strain has recently gained considerable attention as a mammalian model for regenerative wound healing. In our experimental approach, we elucidated the regenerative response of pancreatic acinar cells in these mice following inflammatory injury and mitogenic stimulation.

Our findings revealed a boost of acinar proliferation in MRL/MpJ animals following cerulein-induced acinar damage compared to C57BL/6 control mice. Noteworthy is that the enhanced proliferation in the pancreas seems to be driven by different molecular mechanisms than the ones reported in other regenerating organs. Specifically, a common feature that emerged from transcriptomic studies is that the healing process in MRL/MpJ mice is associated with repression of genes responsible for inflammation^14^. Contrary to these findings, we showed that inflammation was higher in MRL/MpJ pancreas during pancreatic injury. In addition, reduction of inflammatory cell infiltration upon MS-275 treatment did not alter the levels of acinar proliferation, indicating that reduced inflammation is not a pre-requisite for enhanced pancreatic regeneration in MRL/MpJ mice.

Another critical mechanism supporting enhanced proliferation in MRL/MpJ mice is linked to a checkpoint defect in the G1 stage of the cell cycle^19^. In particular, in the ear hole closure model, ear tissue cells did not express the cell cycle inhibitor p21^WAF1/Cip1^ and lack of the protein in p21^-/-^ mice was sufficient to recapitulate this regenerative phenotype. This scenario was not observed in pancreatic tissue, as p21 was highly up-regulated in MRL/MpJ pancreata, together with other cell cycle inhibitor family members, and its deficiency did not result in enhanced proliferation of acinar cells^28^. Moreover, enhanced proliferation in MRL/MpJ dermal cells is associated with an active and constitutive DNA damage check point^19^. However, levels of DNA damage were not higher in acinar cells of MRL/MpJ upon cerulein-induced acinar damage, suggesting that increased proliferation does not depend on regulation of DNA repair mechanisms.

Collectively, the cellular and molecular parameters associated to the “super-healing” ability of several MRL/MpJ cell types were not regulated in a similar manner in pancreatic acinar cells, suggesting the existence of different causative factors in the increased acinar proliferation observed.

An unexpected finding of this study and a likely contributing factor to the proliferative phenotype is that MRL/MpJ mice are more sensitive to cerulein-induced acinar injury compared to C57BL/6 control animals. The increased acinar injury was manifest by higher blood levels of pancreatic enzymes, intra-pancreatic trypsin activation and acinar apoptosis. The causal correlation between enhanced acinar injury and enhanced acinar proliferation is supported by the fact that i) acinar proliferation was still elevated following high injury but decreased inflammation upon MS-275 treatment, while ii) the proliferative advantage was lost upon T3 treatment, which results in minimal acinar injury. This raises the intriguing hypothesis that soluble factors initially released by damaged acinar cells, rather than further damage inflicted by infiltrating inflammatory cells, are key triggers of acinar proliferation. Further analyses are warranted to identify these factors and their “sensors” in intact acinar cells.

Another interesting aspect emerging from our study is the enhanced leukocyte infiltration and expression of pro-inflammatory mediators observed in the pancreas of MRL/MpJ mice. Amongst the different leukocyte populations, we found that macrophages and T cells were recruited extensively to the pancreas after injury. Macrophages can play a dual and opposing role in the development of inflammatory diseases. In a typical example, macrophages have been shown to mediate renal repair in lupus-resistant mice but, conversely, to induce injury in lupus-susceptible MRL-*Fa*s^lpr^ mice and aged MRL/MpJ mice^29^. Macrophages play a pivotal role in the pancreas, where they regulate the progression of inflammation during pancreatitis (reviewed in^30,31^). In addition, they secrete inflammatory cytokines that induce acinar cell reprogramming into ADM^23^. Amongst the different cytokines secreted, RANTES/CCL5 and TNF were identified as the major inducers of ADM events. Importantly, TNF was more expressed in the pancreas of MRL/MpJ mice 72 h after injury. At the same time point, these animals presented a striking increase of ADM. Excessive ADM can be associated with impaired regeneration of pancreatic tissue^32^. However, this was not the case in MRL/MpJ mice, as proliferation of intact acinar cells was not reduced. Increased ADM was rather a consequence of increased inflammation as (i) high ADM levels were reversible and decreased at 96 h concomitantly with decreased leukocyte infiltration in the pancreas and (ii) reducing inflammation with MS-275 treatment also limited ADM formation. In this context it is worth mentioning that MS-275 directly reduced acinar de-differentiation into ADM in acinar explants isolated from C57BL/6 mice^33^, thus we cannot exclude a similar direct effect also in MRL acini.

In conclusion, the results of the present study demonstrate the existence of important differences in the responses of MRL/MpJ mice to pancreatic acinar cell injury compared with C57BL/6 control strain. Specifically, our data provide evidence for enhanced regenerative ability of acinar cells in MRL/MpJ mice in response to increased acinar cell injury, thus prompting the use of MRL/MpJ mice to further investigate the molecular factors capable to stimulate pancreatic regeneration.

In addition, we demonstrated comparable proliferation of pancreatic acini in the two strains upon mitogenic stimulation. This suggests that the molecular mechanisms that control the extent of cell division vary between different types of proliferative triggers in the same tissue. Confirming this concept, the ability of MRL/MpJ mice to regenerate heart tissue was found to depend on the severity of initial injury^9,34^ and wound healing was not enhanced in MRL/MpJ mice when the injury was applied on dorsal skin^35^.

Finally, we showed that MRL/MpJ mice are more sensitive to cerulein-induced damage of acinar cells and develop exacerbated pancreatic inflammation compared with C57BL/6 control strain. These results have the important implication that MRL/MpJ mice can also be exploited as an experimental model for a more severe form of pancreatitis. A greater complement of experimental models with increased severity will allow us to not only better understand the molecular mechanisms of the disease manifestations found in human, where pancreatitis varies from a mild to a life-threatening form aggravated by sepsis and multi-organ failure, but also to devise effective therapeutic approaches.

## Methods

### Animal experiments

All animal experiments were performed in accordance with Swiss Federal animal regulations and approved by the cantonal veterinary office of Zurich. All studies involving animals are reported in accordance with the ARRIVE guidelines for reporting experiments involving animals. Mice used in this study were adult 8–14 week old MRL/MpJ mice (Jackson Laboratories, Bar Harbor, ME, USA) bred in our facility and C57BL/6 (Harlan Laboratories, Itingen, Switzerland) mice. Groups of 4–5 mice were kept in standard individually ventilated cages (IVCs) in a SPF (specific pathogen-free) facility. Food and water was provided ad libitum. Only male mice were used in this study. Pancreatic acinar cell injury was induced via six intraperitoneal (i.p.) injections of 50 μg/kg cerulein, administered hourly on two consecutive days. Mice were anesthetized by isoflurane inhalation and harvested over a period of 96 h. The harvest times for the different experiments are expressed as hours after the first cerulein injection and are specified in the individual figure panels. Class I HDAC inhibitor MS-275 (Selleckchem, Houston, USA) was injected daily i.p. at 20 mg/kg, starting one day before the first cerulein injection. Control animals received vehicle DMSO injections. 3,5,3-L-tri-iodothyronine (T3) was administered daily i.p. at 400 mg/kg. Control animals received vehicle 0.1 M NaOH, pH 7.4 with 0.5%BSA injections.

Groups of 5 animals were tested for each experiment. Animals were assigned randomly to different experimental groups for all *in vivo* studies. Data collection and evaluation of all experiments were performed blinded to the group identity.

### Biochemical analyses of enzyme activity

Levels of enzymatic activity of amylase, lipase, glutamate pyruvate transaminase (GPT) and glutamic oxaloacetic transaminase (GOT) were measured in blood serum collected via heart puncture. Enzymes were measured using the Fuji Dri-Chem 4000i analyzer (FUJIFILM Corporation, Tokyo, Japan).

Trypsin activity was measured fluorometrically using Boc-Gln-Ala-Arg-AMC as a substrate, according to the method of Kawabata *et al*.^36^.

For the myeloperoxidase assay, lung samples were homogenized in 0.5% hexadecyltrimethylammonium bromide and enzymatic assay was measured using o-dianisidine dihydrochloride as a substrate, as previously described^37^.

### Detection of autoantibodies against pancreatic juice proteins

Microtiter plates were coated with 5 μg/mL of rat pancreatic juice, in 50 mM sodium carbonate buffer (pH 9.5). After blocking with 5% non-fat milk, each well was incubated with mouse serum samples diluted 1:10, followed by incubation with alkaline phosphatase conjugate goat anti-mouse IgG (Sigma, Buchs, Switzerland). Bound antibodies were detected with phosphatase substrate (Sigma, Buchs, Switzerland) using an ELISA plate reader (Dynex MRXII, Molecular Devices, San Jose, USA).

### Transcript analysis

Total RNA was extracted from pancreatic tissue, as described previously^38^, and RNA quality control was performed by RIN (RNA Integrity Number) measurement using a 2100 Bioanalyzer system (Agilent, Santa Clara, CA, USA). RNA was reverse-transcribed with qScript™ cDNA SuperMix (Quanta Biosciences, Beverly, CA, USA). Gene expression was measured by real-time PCR on a 7500 Fast Real-Time PCR System (Applied Biosystems, Foster City, USA) using Taqman probes (Applied Biosystems, Foster City, CA, USA). Transcript levels were normalized using 18S RNA as a reference and expressed as 2^−ΔΔCt^ relative to the value of control animals. Primer probes used were the following: Ccna2 (Mm00438064_m1), Ccnb1 (Mm01171453_m1), Ccnd1 (Mm00432359_m1), Ccne1 (Mm00432367_m1), Cdkn1a (p21) (Mm00432448_m1), Cdkn2a (p16) (Mm00494449_m1), Cdkn1b (p27) (Mm00438168_m1), Trp53 (p53) (Mm01731287_m1), Sox9 (Mm00448840_m1), Hes1 (Mm01342805_m1), Krt19 (Mm00492980_m1), Amy1 (Mm00651524_m1), Mist1 (Mm00487695_m1), Ptf1a (Mm00479622_m1), F4/80 (Mm00802530_m1), Cd40 (Mm00441891_m1), Nos2 (Mm00440502_m1), Arg1 (Mm00475988_m1), Mrc1 (Mm00485148_m1), Cd3 (Mm00446171_m1), Gata 3 (Mm00484683_m1), Tbet (Mm00450960_m1), Il6 (Mm00446190_m1), Il1b (Mm00434228_m1), Cox2 (Mm00478374_m1), Tnf (Mm00443258_m1), Ccl5 (Mm01302427_m1), Ccl2 (Mm00441242_m1), Cxcl1 (Mm04207460_m1), Cxcl2 (Mm00436450_m1), Cxcl16 (Mm00469712_m1), Cckar (Mm00438060_m1), Cckbr (Mm00432329_m1), TaqMan^TM^ ribosomal RNA control reagent (#4308329). Rorgt was expression was quantified using SYBR^TM^ Green One-Step qRT-PCR Master Mix (Applied Biosystems, Foster City, CA, USA) with the primers F: CACGGCCCTGGTTCTCAT; R: GCAGATGTTCCACTCTCCTCTTCT.

### Immunohistochemistry

Pancreas specimens were embedded in paraffin for histological analyses, as described^39^. Primary antibodies used in this study were: rabbit anti-Ki67 (#ab16667, Abcam, Cambridge, UK, 1:200); rabbit anti-amylase (#A8273-1VL, Sigma-Aldrich, Buchs, Switzerland, 1:1000); rabbit anti-PU.1 (#2266, Cell Signaling Technologies, Danvers, MA, 1:200); rabbit anti-phospho-histone H2A.X (Ser139) (#9718, Cell Signaling Technologies, Danvers, MA, 1:500); rat anti-F4/80 (#T-2006 BMA Biomedicals, Augst, Switzerland, 1:100); rabbit anti-Cd3 (#A0452, Dako, Santa Clara, CA, 1:150); rabbit anti-cleaved Caspase-3 (Asp175) (#9661, Cell Signaling Technologies, Danvers, MA, 1:1500). Secondary antibodies used in this study were biotinylated goat anti-rabbit IgG (H + L), included in the Vectastain® ABC Kit (PK-4001, Vector Laboratories, Peterborough, UK). Nuclei were visualized with 4′,6-diamidino-2-phenylindole (DAPI). All antibodies are routinely used in our laboratory.

For quantitative analysis of intact acinar tissue, paraffin-embedded pancreas specimens were immunostained for amylase, slides were scanned with a NDP NanoZoomer Digital Pathology Slide Scanner (Hamamatsu, Hamamatsu City, Japan) and analyzed in a blinded fashion. The area occupied by amylase-positive acinar tissue was expressed as percentage of total pancreatic area present in each slide.

Microscopy analyses were performed on a wide-field Nikon Eclipse Ti (Amsterdam, The Netherlands). Quantification of labelled cells was performed in at least 10 randomly selected high-power fields (×200) per slide using the NIS Elements BR Analysis and Cell^P analysis software. Number of positive cells was normalized on the area occupied by pancreatic acinar cells present in each power field. Pancreatic ducts, islets and vessels were excluded from the analysis.

### Statistics

Groups of 5 animals were tested for each experimental group. The data are expressed as means ± SEM. The statistical significance of differences in the means of experimental groups was determined using an unpaired, two-tailed Student’s *t* test or one-way analysis of variance followed by Dunnett post test (GraphPad Prism 4.0c; GraphPad Software, Inc.) and a probability value <0.05 was considered statistically significant.

### Data availability statement

The data generated during the current study are available from the corresponding author on reasonable request.

## Electronic supplementary material

Supplementary figures
