# Supplementary material for: Enhanced proliferation of pancreatic acinar cells in MRL/MpJ mice is driven by severe acinar injury but independent of inflammation
Source: Sci Rep. 2018 Jun 20;8:9391. doi: 10.1038/s41598-018-27422-0 (PMC6010442; doi:10.1038/s41598-018-27422-0)
Supplement: Supplementary file 1 — Supplementary figures [file 41598_2018_27422_MOESM1_ESM.pdf]

Enhanced proliferation of pancreatic acinar cells in MRL/Mpj mice is driven by severe acinar injury but independent of inflammation.

Marta Bombardo[1], Ermanno Malagola[1], Rong Chen[1], Arcangelo Carta[1], Gitta M. Seleznik[1], Andrew P. Hills[3], Rolf Graf[1,2] and Sabrina Sonda[1,2,3*]

[1]Swiss Hepato-Pancreato-Biliary Center, Department of Visceral and Transplantation Surgery, University Hospital, Zurich, Switzerland; [2]Center for Integrative Human Physiology (ZIHP), University of Zurich, Switzerland. [3]School of Health Sciences, College of Health and Medicine, University of Tasmania, Australia.

SUPPLEMENTARY FIGURES

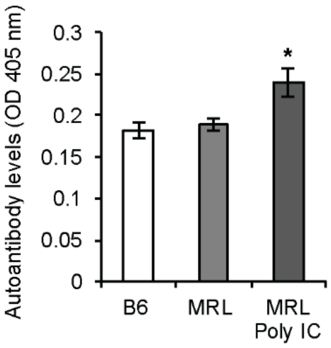

Figure S1

Supplementary figure 1. Quantification of autoantibodies present in pancreatic juice of 14 week old C57BL/6 (B6) and MRL/MpJ (MRL) mice. Pancreatic juice of MRL mice treated with polyinosinic polycytidylic acid (poly IC) were used as positive control for autoantibody production. Results are average ± SEM (n=5), *P < 0.05.

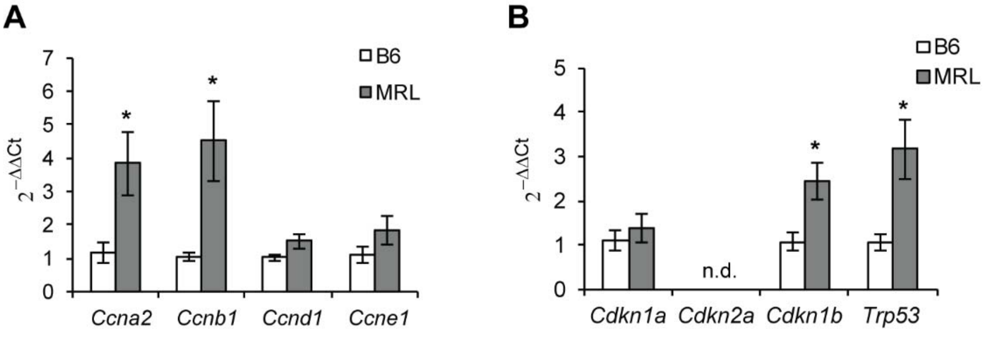

Figure S2

Supplementary figure 2. qPCR of cyclins (**A**) and CDK inhibitors and p53 (**B**) in pancreata of untreated C57BL/6 (B6) and MRL/MpJ (MRL) mice. N.d., not detectable. Results are average ± SEM (n=5).

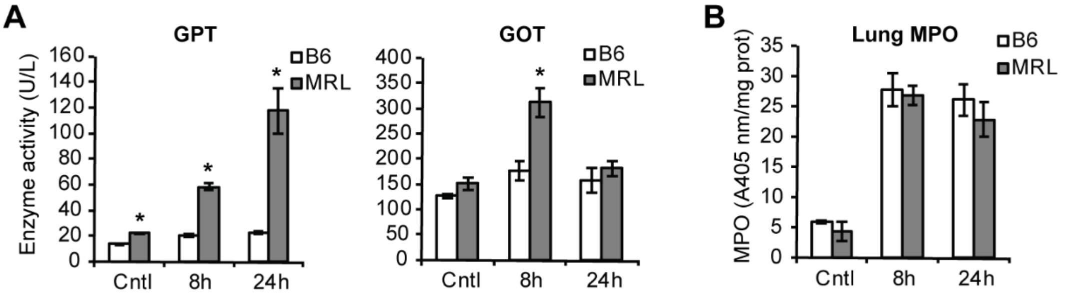

Figure S3

Supplementary figure 3. (**A**) Serum levels of glutamate pyruvate transaminase (GPT) and glutamic oxaloacetic transaminase (GOT) in C57BL/6 (B6) and MRL/MpJ (MRL) mice in control (Cntl) animals and at the indicated time after cerulein administration. (**B**) Myeloperoxidase (MPO) activity in lungs in control (Cntl) animals and at the indicated time after cerulein administration. Results are average ± SEM (n=5), *P < 0.05.

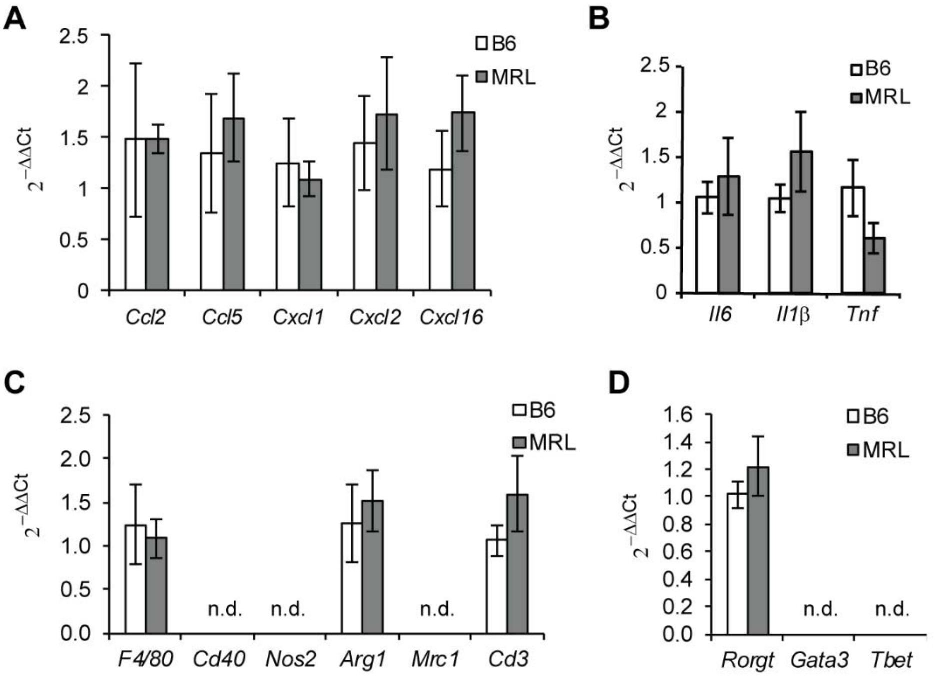

Figure S4

Supplementary figure 4. qPCR of chemokines (**A**), cytokines (**B**), macrophage and T cell markers (C), and T cell transcription factors (D) in pancreata of untreated C57BL/6 (B6) and MRL/MpJ (MRL) mice. N.d., not detectable. Results are average ± SEM (n=5), *P < 0.05.

.

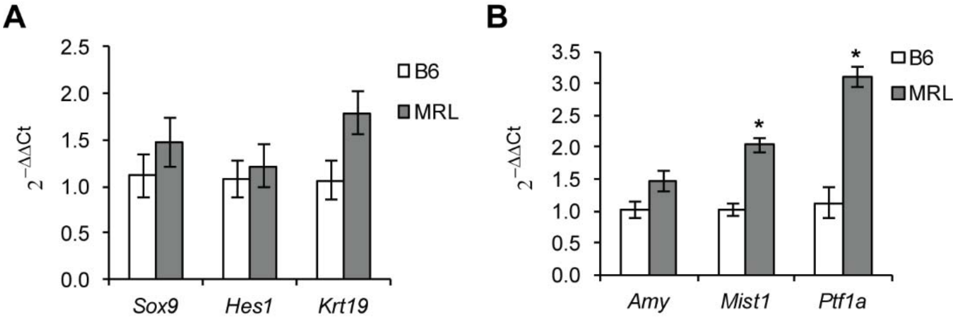

Figure S5

Supplementary figure 5. qPCR of progenitor (**A**) and differentiation genes (**B**) in pancreata of untreated C57BL/6 (B6) and MRL/MpJ (MRL) mice. Results are average ± SEM (n=5), *P < 0.05.

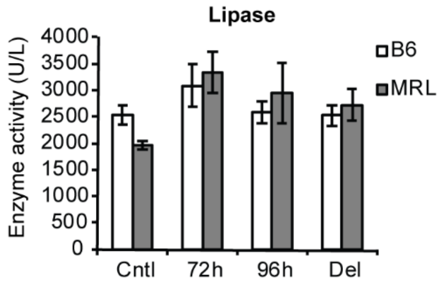

Figure S6

Supplementary figure 6. Serum levels of lipase enzymatic activity in C57BL/6 (B6) and MRL/MpJ (MRL) mice in control (Cntl) animals and at the indicated time after T3 administration. Del, delayed model. Results are average ± SEM (n=5).
